# Hypoxia-regulated non-coding RNAs in OSCC liquid biopsy: from molecular mechanisms to AI-driven precision oncology

**DOI:** 10.3389/fonc.2026.1902907

**Published:** 2026-08-17

**Authors:** Pooja Singh, Manoj Pandey

**Affiliations:** 1 Department of Surgical Oncology, Institute of Medical Sciences, Banaras Hindu University, Varanasi, India; 2 DHR-ICMR Advanced Molecular Oncology Diagnostic Services (DIAMOnDS), Institute of Medical Sciences, Banaras Hindu University, Varanasi, India

**Keywords:** artificial intelligence, exosomes, hypoxia, liquid biopsy, non-coding RNAs, oral squamous cell carcinoma, point-of-care

## Abstract

**Background:**

Oral squamous cell carcinoma (OSCC) is characterized by late-stage diagnosis and high recurrence rates. Intratumoral hypoxia, driven by hypoxia-inducible factors 1α and 2α (HIF-1α/HIF-2α), orchestrates aggressive phenotypes including epithelial–mesenchymal transition (EMT), metabolic reprogramming, stemness maintenance, and therapeutic resistance. Non-coding RNAs (ncRNAs) serve as central post-transcriptional regulators of these hypoxic adaptations and offer promising non-invasive biomarker potential.

**Methods:**

A systematic literature search of the PubMed database was executed on May 15, 2026, using structured Boolean combinations of Medical Subject Headings (MeSH) terms and high-accuracy text keywords targeting OSCC, hypoxia pathways (HIF-1α/HIF-2α), non-coding RNA classes (miRNAs, lncRNAs, circRNAs), and liquid biopsy media (saliva, plasma, serum, exosomes).

**Results:**

Hypoxic stress stabilizes HIF-1α during acute oxygen deprivation and HIF-2α during chronic adaptation, driving widespread dysregulation across the non-coding transcriptome. HIF-1α-activated “hypoxemirs” (e.g., miR-210) and oncogenic lncRNAs (MALAT1, H19, HOTAIR) coordinate early glycolytic switching and invasive EMT cascades. Conversely, chronic HIF-2α activation regulates specific downstream targets (e.g., lncRNA RAB11B-AS1, NEAT1) linked to persistent microvascular remodeling and cancer stem cell preservation. Closed-loop circular RNAs (e.g., circCDR1as, hsa-circ-0001030) display enhanced structural resistance to exonuclease degradation and act as molecular sponges within competing endogenous RNA (ceRNA) networks. When encapsulated within extracellular vesicles (exosomes) or secreted into saliva and blood, these stable transcripts provide a longitudinal window into tumor microenvironment dynamics. Incorporating artificial intelligence (AI) machine learning models improves multi-transcript signature filtering, while microfluidic point-of-care (POC) devices offer a practical roadmap for decentralized diagnostic deployment.

**Conclusion:**

Hypoxia-responsive ncRNAs isolated from liquid biopsies represent promising candidates for OSCC precision oncology. However, routine clinical translation remains restricted as most candidate biomarkers are currently in Phase 1 discovery or Phase 2 early biofluid validation stages. Widespread clinical adoption is limited by pre-analytical sample handling variability, a lack of universally stable internal reference controls, cohort heterogeneity, insufficient prospective multi-center validation, and regulatory approval barriers.

## Graphical Abstract

**Figure:**
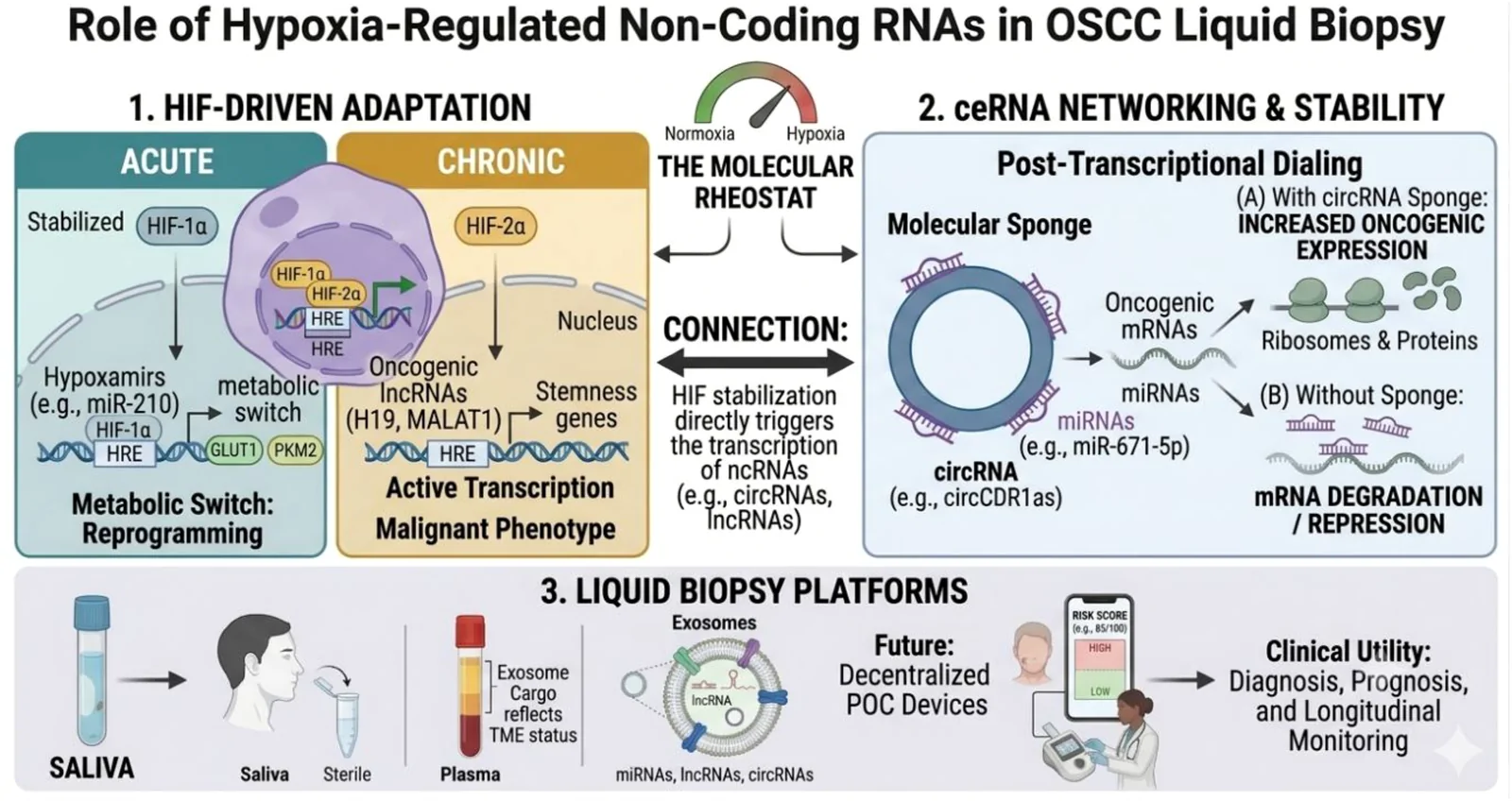
Role of hypoxia-regulated non-coding RNAs in OSCC liquid biopsy. A schematic representation showing: (1) HIF-Driven Adaptation, illustrating acute (HIF-1α) metabolic switching versus chronic (HIF-2α) malignant transactivation; (2) ceRNA Networking & Stability, showing circular RNAs acting as molecular sponges to prevent miRNA-mediated mRNA degradation, resulting in increased oncogenic protein expression; and (3) Liquid Biopsy Platforms, mapping the isolation of circulating miRNAs, lncRNAs, and circRNAs from saliva, blood plasma, and exosomal cargo for future point-of-care (POC) clinical risk scoring, early diagnosis, prognosis, and longitudinal therapeutic monitoring.

## 1 Introduction

### 1.1 The clinical challenge of OSCC

Oral squamous cell carcinoma (OSCC) accounts for nearly 90% of oral malignancies and represents a major global health burden (1). Despite significant improvements in surgical techniques, radiotherapy, chemotherapy, and targeted clinical regimens, the five-year survival rate of OSCC patients remains relatively poor (2). This diagnostic failure is primarily driven by the fact that a vast majority of patients present at advanced stages characterized by occult cervical lymph node metastasis and structural resistance to standard therapies (2). Consequently, the identification of highly reliable, minimally invasive molecular biomarkers for early detection and active risk stratification remains a paramount challenge in clinical oncology (3–5).

### 1.2 Hypoxia and the tumor microenvironment

Hypoxia is a foundational, pathognomonic characteristic of the solid tumor microenvironment (TME) across solid malignancies, including aggressive oral cancers (6, 7). Rapid, unchecked cellular proliferation outpaces localized angiogenesis, creating severe oxygen-deficient niches within the structural architecture of the tumor (7). Cellular adaptation to this hypoxic stress is fundamentally orchestrated by hypoxia-inducible factors (HIFs), which act as master transcription factors regulating an array of genes involved in structural angiogenesis, metabolic glycolysis, active cellular proliferation, distal metastasis, and overall cell survival (8). Elevated expression of HIF-1α in OSCC has been consistently correlated with poor overall survival, accelerated invasion patterns, and direct resistance to standard chemoradiotherapy regimens (9). Accumulating biological evidence highlights a functional segregation between the primary HIF alpha isoforms based on the duration of oxygen deprivation: HIF-1α primarily mediates the acute hypoxic response, acting as the dominant transcriptional driver of immediate glycolytic switching, metabolic reprogramming, and early microvascular sprouting (10, 11). HIF-2α accumulates predominantly during sustained, chronic hypoxia, directing long-term cellular adaptation, cancer stem cell (CSC) phenotype maintenance, and structural extracellular matrix remodeling (10–12).

### 1.3 Non-coding RNAs as liquid biopsy biomarkers

Recent high-throughput transcriptomic insights have highlighted the pivotal regulatory roles played by non-coding RNAs (ncRNAs) within this hypoxia-mediated signaling network (13). ncRNAs are a class of functional RNA molecules that lack classical protein-coding capacity; they are broadly categorized into:

- MicroRNAs (miRNAs): Short, single-stranded transcripts involved in post-transcriptional gene silencing (13).
- Long non-coding RNAs (lncRNAs): Transcripts longer than 200 nucleotides that act as epigenetic and transcriptional scaffolds (13).
- Circular RNAs (circRNAs): Covalently closed, continuous loop configurations displaying high stability (13).

**Figure 1:**
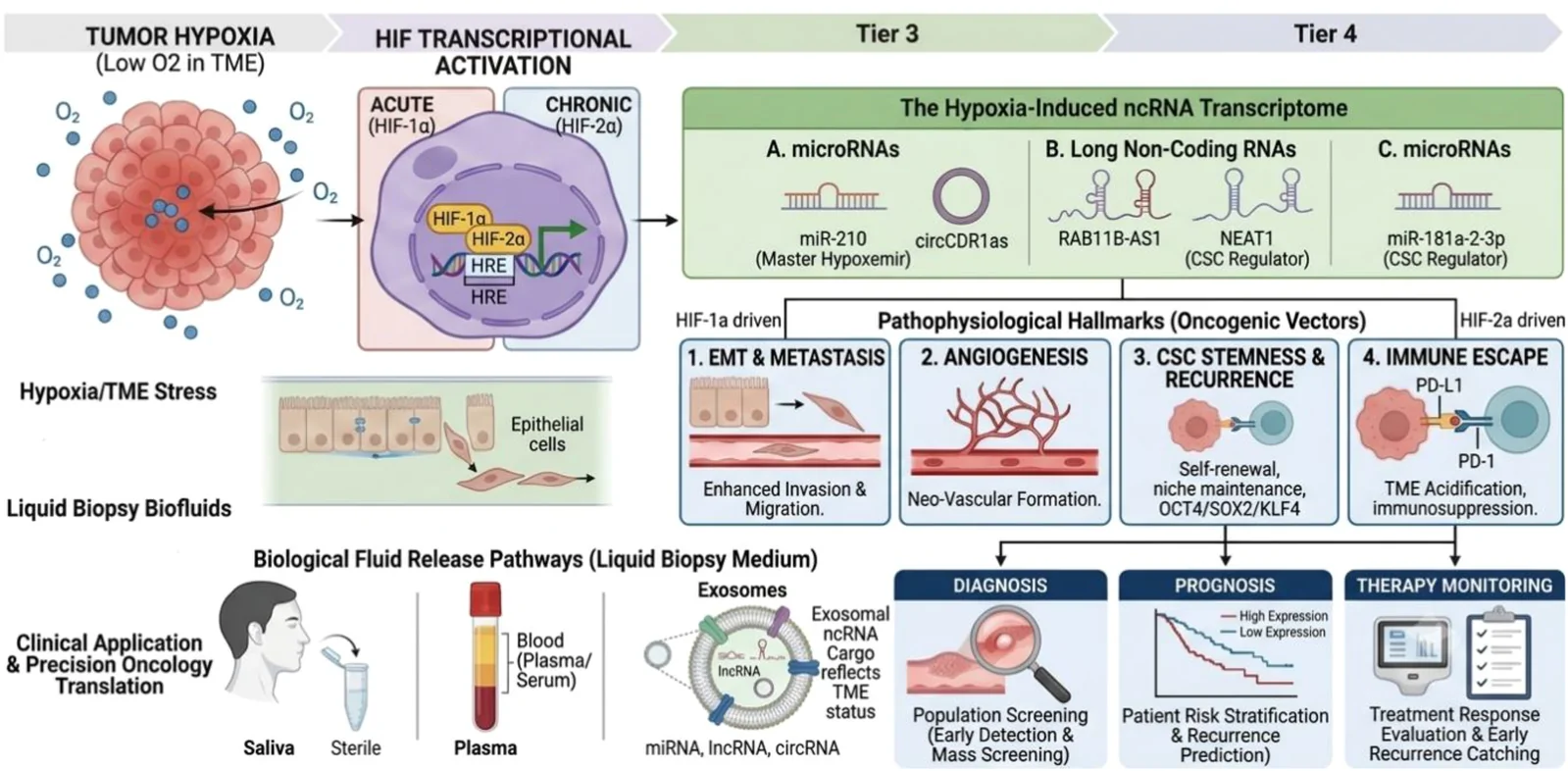
The mechanistic cascade of acute vs. chronic hypoxia-induced non-coding RNAs (ncRNAs) as liquid biopsy biomarkers in OSCC. A multi-tier hierarchical overview tracing the pipeline from restricted tumor microenvironment (TME) oxygenation to differential HIF transcriptional activation. The diagram explicitly contrasts acute HIF-1α responses (driving miR-210, early EMT, and glycolytic switching) with chronic HIF-2α responses (driving lncRNA RAB11B-AS1, lncRNA NEAT1, miR-181a-2-3p, cancer stem cell stemness preservation, and immune escape). These dysregulated ncRNA species are released via saliva, blood plasma/serum, and exosomal cargo, providing stable biofluid targets for population screening, patient risk stratification, and longitudinal treatment monitoring.

When dysregulated by hypoxic stress, these varied ncRNA species influence multiple downstream pathways associated with oral carcinogenesis, extracellular matrix remodeling, and immune evasion (13). Crucially, because these molecules are actively shed from tumor cells into accessible biofluids; such as saliva, plasma, and serum, they provide a highly attractive, minimally invasive liquid biopsy medium to execute longitudinal disease monitoring (14, 15) (**Figure 1**).

### 1.4 Literature context, novelty, and review scope

While several narrative reviews exist on general non-coding RNAs in oral cancer, salivary ncRNAs, exosomal ncRNAs, or liquid biopsy in OSCC (13–15), a key literature gap remains regarding the biological intersection of hypoxia-induced TME stress and non-coding RNA dynamics specifically in liquid biopsy applications. Hypoxia-regulated ncRNAs (“hypoxemirs”, oncogenic lncRNAs, and closed-loop circRNAs) warrant dedicated focus because hypoxia is the primary microenvironmental driver of aggressive phenotypes, and these specific stress-induced transcripts possess unique functional roles and enhanced stability profiles in circulating biofluids. This review systematically synthesizes the mechanistic role of hypoxia-regulated ncRNAs in OSCC and evaluates their translational potential as liquid biopsy biomarkers. To ensure scientific rigor and clear structural boundaries this review provide an evidence-based synthesis evaluating subcellular molecular signaling, structural transcript stability, and candidate ncRNA performance across tissue and biofluid media and an evidence-informed, forward-looking roadmap examining how multi-omic machine learning signature filtering and microfluidic point-of-care devices can overcome analytical complexity and facilitate decentralized clinical testing.

## 2 Methods

### 2.1 Search strategy and bibliographic sources

To ensure full methodological transparency and reproducibility, a systematic literature search was executed on the PubMed database on May 15, 2026. The bibliographic search targeted all peer-reviewed studies published up to the search execution date investigating non-coding RNAs, hypoxia signaling pathways, and liquid biopsy modalities in oral squamous cell carcinoma. Following established narrative synthesis guidelines (13), the literature query was structured using a comprehensive Boolean combination of Medical Subject Headings (MeSH) and high-accuracy text keywords: ((“Oral Squamous Cell Carcinoma”[Mesh] OR “OSCC” OR “Head and Neck Squamous Cell Carcinoma” OR “HNSCC”) AND (“Hypoxia”[Mesh] OR “HIF-1alpha” OR “HIF-2alpha” OR “Hypoxia-Inducible Factor”) AND (“MicroRNAs”[Mesh] OR “miRNA” OR “RNA, Long Noncoding”[Mesh] OR “lncRNA” OR “RNA, Circular”[Mesh] OR “circRNA” OR “non-coding RNA”) AND (“Liquid Biopsy”[Mesh] OR “Exosomes”[Mesh] OR “Saliva”[Mesh] OR “Plasma” OR “Serum” OR “Circulating Biomarkers”)) Only full-text, peer-reviewed original research articles, mechanistic evaluations, and clinical biomarker studies indexed within the PubMed database were retrieved for screening.

### 2.2 Study selection and eligibility criteria

Retrieved literature underwent multi-tier screening against predefined inclusion and exclusion parameters to eliminate selection bias. Studies were considered eligible if they evaluated hypoxia-associated or hypoxia-responsive non-coding RNA transcripts (miRNAs, lncRNAs, or circRNAs) within oral squamous cell carcinoma or head and neck squamous cell carcinoma models. Eligible studies were required to report definitive mechanistic data regarding tumor adaptation (e.g., EMT, metabolic switching, stemness, immune escape) or report clinical performance parameters (such as ROC-AUC, sensitivity, specificity, hazard ratios, or cohort survival metrics) across specific biological matrices including cell lines, primary tumor tissues, whole saliva, blood plasma, serum, or isolated exosomal fractions. Conversely, studies were systematically excluded if they lacked specific hypoxia or HIF-dependent functional endpoints, investigated non-oral malignancies without translational relevance to OSCC/HNSCC, lacked peer-reviewed full-text articles (e.g., conference abstracts, editorials, pre-prints), or represented duplicate publications.

### 2.3 Data extraction and quality assessment

Data from eligible publications were manually extracted into a standardized documentation matrix. Extracted parameters comprised primary author details, publication year, experimental design (*in vitro* cell line, animal model, tissue cohort, or prospective/retrospective biofluid study), specific ncRNA variant and transcript class, targeted molecular signaling pathway, biological matrix evaluated, quantitative detection platform (e.g., RT-qPCR, ddPCR, NGS, microarray), and reported clinical utility metrics. Study quality and methodological rigor were assessed dynamically by examining sample size adequacy (n), cohort demographic representation, biological reproducibility, assay normalization controls, and validation status (Phase 1 pre-clinical discovery versus Phase 2 biofluid clinical validation). Particular attention was directed toward distinguishing studies conducted strictly *in vitro* or in tissue specimens from those performing true circulating biofluid evaluations.

### 2.4 Data synthesis and molecular pathway mapping

Due to the substantial clinical and technical heterogeneity across included studies; comprising divergent sample matrices, variable exosome isolation SOPs, and non-standardized internal reference controls, a quantitative meta-analysis was not methodologically feasible. Instead, a non-statistical narrative synthesis framework was implemented following established guidance for mapping complex molecular pathways (16–18). This synthesis integrated molecular cross-talk across major pathophysiological hallmarks, tracing how hypoxia-inducible factors drive specific ncRNA expression to modulate epithelial–mesenchymal transition, GLUT1/PKM2-mediated glycolytic switching, angiogenesis, and exosomal immune evasion. Furthermore, the synthesis integrated translational paradigms by evaluating multi-omic machine learning signature filtering alongside decentralized microfluidic point-of-care biosensors to map the clinical pipeline from initial discovery to bedside deployment.

## 3 Results and discussion

### 3.1 Molecular foundations of the HIF-ncRNA axis and temporal selectivity

**Figure 2:**
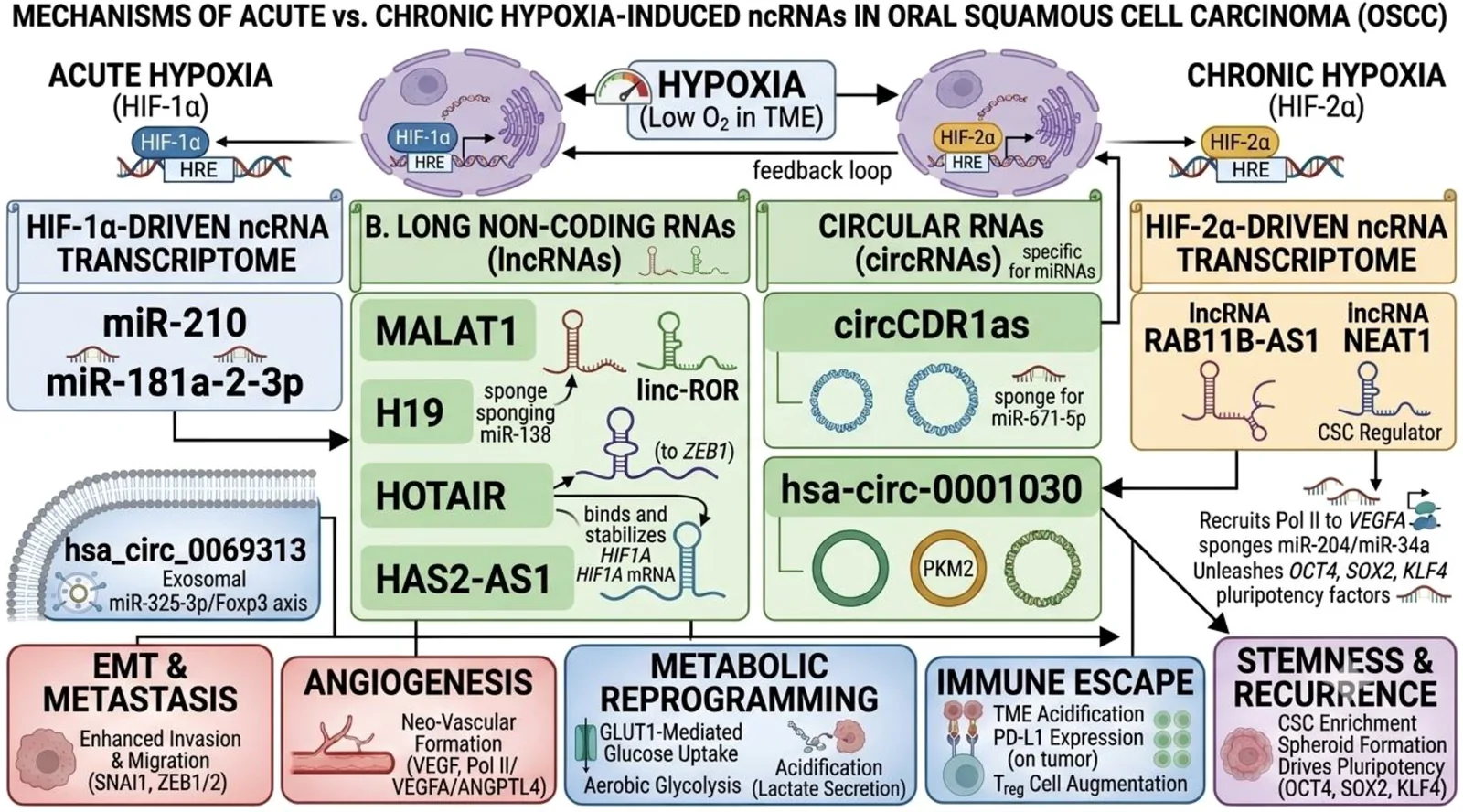
Mechanisms of acute vs. chronic hypoxia-induced ncRNAs in oral squamous cell carcinoma (OSCC). A detailed molecular schematic illustrating specific mechanistic targets of hypoxia-induced non-coding RNAs. The acute HIF-1α axis drives early metabolic switching, invasion, and matrix remodeling via miR-210, MALAT1, H19, HOTAIR, and HAS2-AS1. The chronic HIF-2α axis regulates distinct non-coding transcripts—including lncRNA RAB11B-AS1 and lncRNA NEAT1—to promote microvascular remodeling, nuclear paraspeckle assembly, and pluripotency factors (OCT4, SOX2, KLF4) involved in cancer stem cell (CSC) preservation and recurrence. Additional vectors depict exosomal hsa_circ_0069313 driving TME immune escape via the miR-325-3p/Foxp3/PD-L1 signaling axis and metabolic regulation by hsa-circ-0001030.

The adaptive capacity of oral squamous cell carcinoma (OSCC) under restricted oxygenation is fundamentally coordinated through temporal, isoform-specific transcription factor dynamics (6, 7). Under baseline normoxic conditions, hypoxia-inducible factor alpha (HIFα) subunits undergo rapid oxygen-dependent hydroxylation mediated by prolyl hydroxylase domain enzymes (PHDs), targeting them for von Hippel–Lindau (VHL)-mediated ubiquitination and 26S proteasomal degradation (6, 7). However, restricted vascularization within the tumor microenvironment (TME) limits oxygen availability, suppressing PHD activity and permitting stabilized HIFα subunits to accumulate, translocate to the nucleus, dimerize with HIF-1β, and bind hypoxia response elements (HREs) to drive transcriptomic program activation (6, 7). Accumulating biological evidence highlights a functional segregation between the primary HIF alpha isoforms based on the duration of oxygen deprivation (10–12). HIF-1α primarily orchestrates the acute hypoxic response within the first 0 to 24 hours, serving as the dominant transcriptional driver of immediate glycolytic switching, metabolic reprogramming, and early microvascular sprouting (10, 11). A central effector of this acute axis is the direct transactivation of master “hypoxemirs,” most notably miR-210, which suppresses mitochondrial respiration and facilitates microvascular angiogenesis (19–21)(**Figure 2**).

In contrast, HIF-2α (encoded by EPAS1) accumulates during sustained, chronic hypoxia exceeding 24 to 48 hours to coordinate long-term cellular adaptation, extracellular matrix restructuring, and cancer stem cell (CSC) niche preservation (10–12). Specific non-coding RNA networks operate downstream of or in feedback loops with HIF-2α during this chronic adaptation phase (10, 22, 23). Under prolonged oxygen deprivation, HIF-2α directly binds the promoter region of lncRNA RAB11B-AS1, upregulating its expression to recruit RNA Polymerase II to pro-angiogenic targets such as VEGFA and ANGPTL4, thereby driving sustained microvascular remodeling (10, 23). Furthermore, chronic hypoxic activation of the miR-181a-2-3p regulatory loop selectively stabilizes HIF-2α protein independently of HIF-1α, transactivating core stemness transcription factors including OCT4, SOX2, and KLF4 to enhance self-renewal capacity in OSCC stem-like populations (18, 19). Additionally, persistent HIF-2α activity during chronic hypoxia drives the expression of lncRNA NEAT1, which promotes nuclear paraspeckle assembly and functions as a competitive endogenous RNA (ceRNA) sponge for miR-204-5p and miR-34a-5p to preserve stemness phenotypes and confer chemoresistance (22, 23). While localized exceptions exist—such as floor-of-mouth carcinomas where early HIF-1α expression uniquely indicated favorable outcomes (24); extensive clinical profiling confirms that systemic stabilization of HIF-1α and HIF-2α in advanced OSCC strongly correlates with increased aggressiveness, cervical lymph node metastasis, therapeutic resistance, and reduced overall survival (9, 10).

### 3.2 Mechanistic profiles of hypoxia-induced lncRNAs and regulated cascades

**Figure 3:**
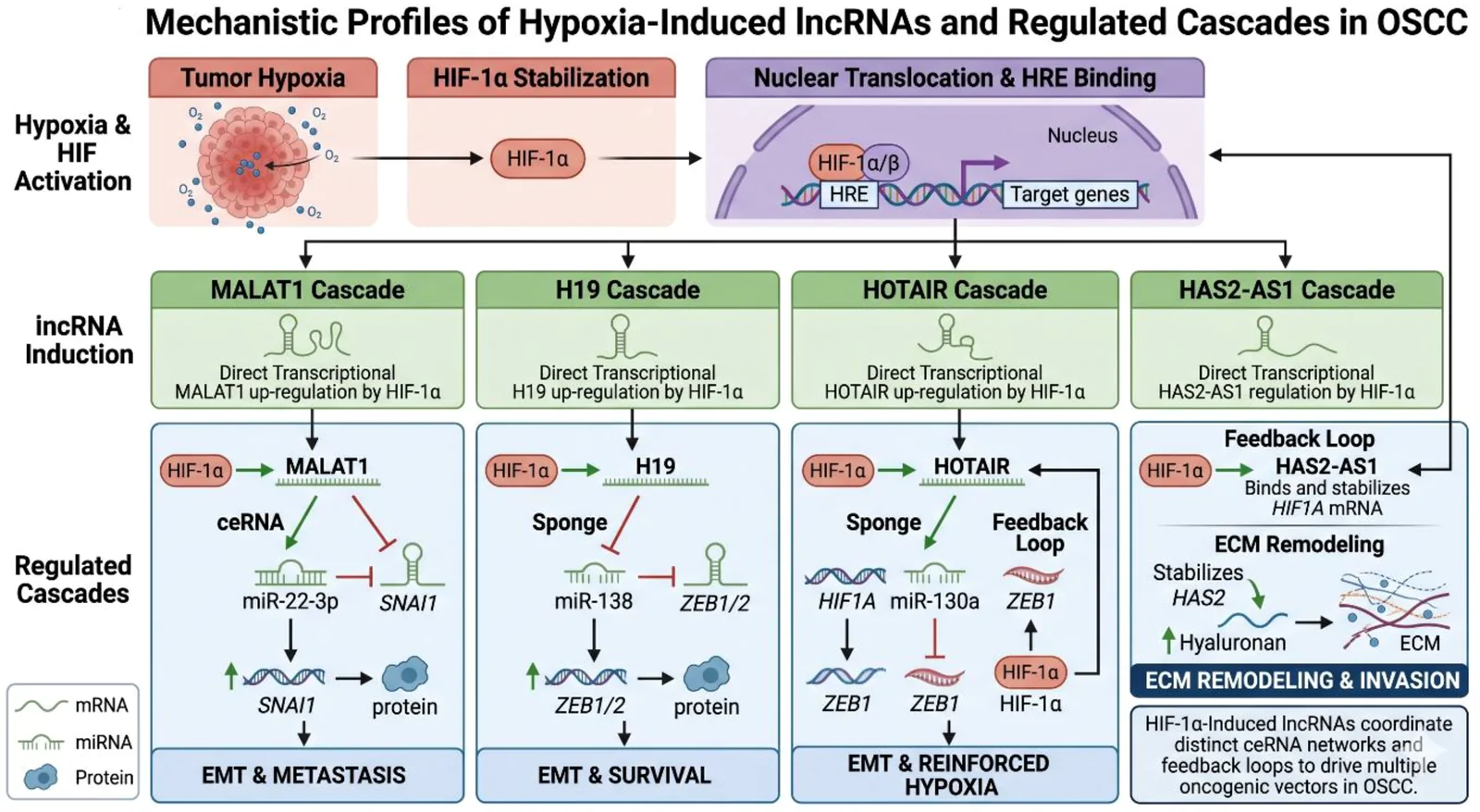
Mechanistic profiles of hypoxia-induced long non-coding RNAs (lncRNAs) and regulated cascades in OSCC. Detailed schematic illustrating four distinct hypoxia-induced lncRNAs in oral cancer. Tumor hypoxia triggers the stabilization and nuclear translocation of HIF-1α, driving direct transcriptional up-regulation of MALAT1, H19, HOTAIR, and HAS2-AS1. Subsequent regulated cascades detail how these lncRNAs coordinate complex competing endogenous RNA (ceRNA) networks and feedback loops to regulate downstream target genes (SNAI1, ZEB1/2, HAS2), driving distinct pathophysiological vectors of EMT, metastasis, cell survival, and extracellular matrix (ECM) remodeling.

Long non-coding RNAs (lncRNAs), defined as functional non-protein-coding transcripts exceeding 200 nucleotides, function as dynamic epigenetic scaffolds and transcriptional regulators that are heavily dysregulated within the hypoxic TME (22, 23). Among these, Metastasis Associated Lung Adenocarcinoma Transcript 1 (MALAT1) represents a well-characterized oncogenic mediator in oral malignancies (25). Hypoxic stress directly upregulates MALAT1, which subsequently triggers the induction of epithelial–mesenchymal transition (EMT) to accelerate cell migration and metastatic spread (26). Downstream mechanistic evaluations demonstrate that MALAT1 activates the Wnt β-catenin signaling loop, effectively suppressing apoptotic cascades to promote cell survival in tongue squamous cell carcinoma (26). Parallel oncogenic cascades are driven by the hypoxia-responsive lncRNA H19 (27). Epigenetic profiling reveals that hypoxia induces hypomethylation of the H19 promoter region, resulting in sustained overexpression in advanced OSCC lesions (27). This hypomethylation-driven overabundance directly correlates with advanced primary tumor stage, lymph node involvement, and shortened patient survival by allowing H19 to act as a ceRNA that sequesters tumor-suppressive microRNAs (27) (**Figure 3**). Similarly, HOX Transcript Antisense RNA (HOTAIR) is upregulated under low-oxygen conditions, where its expression tracks with lymph node metastasis, local proliferation, and therapeutic resistance (25, 28). Additionally, lncRNA HAS2-AS1 is robustly induced by hypoxia to promote cell invasion and directional migration by directly modulating extracellular matrix remodeling pathways and EMT mechanics (29).

### 3.3 The ceRNA network and circular RNAs as molecular sponges

**Figure 4:**
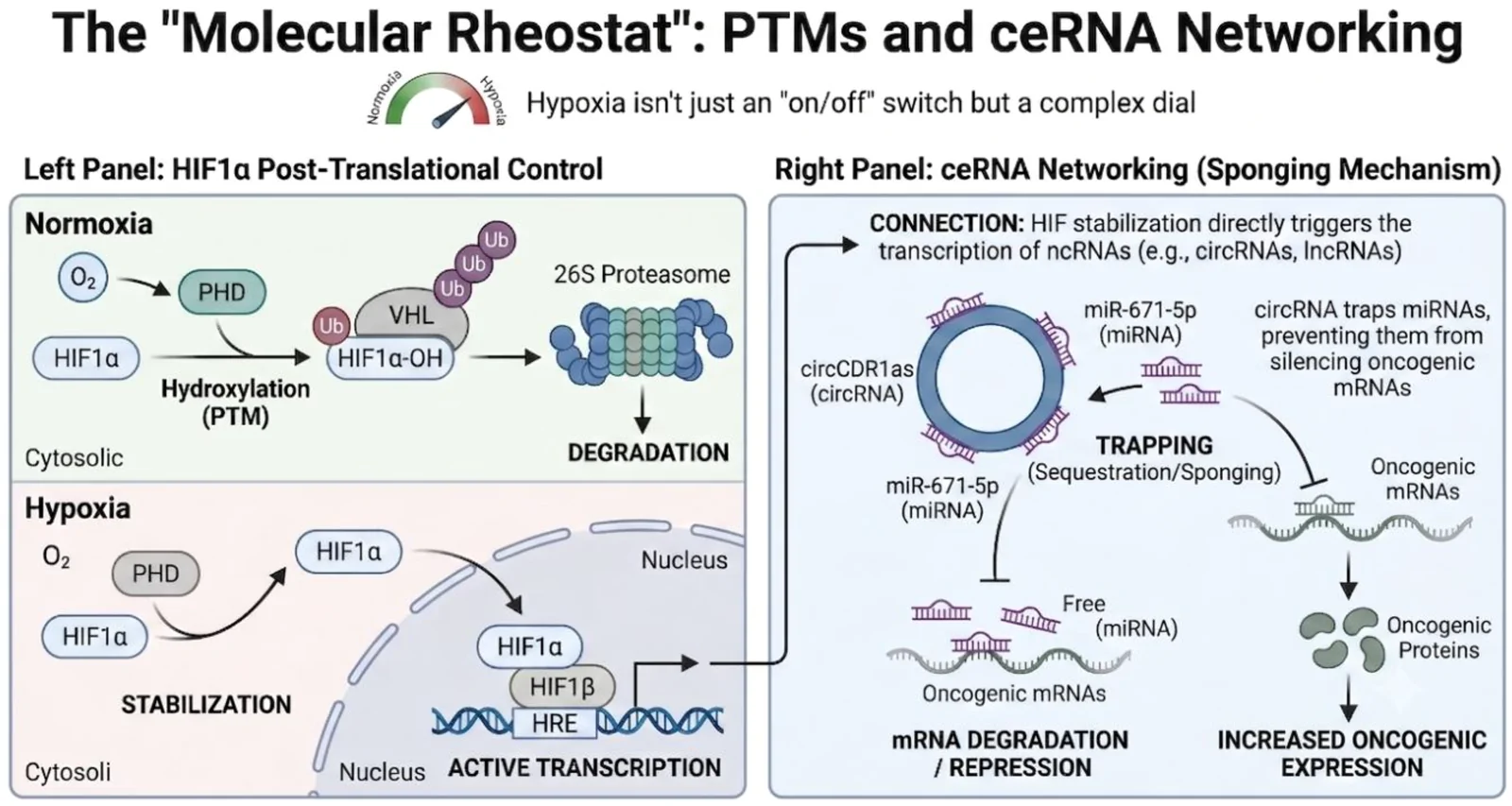
Post-translational control of HIF-1α stabilization and ceRNA network modulation. The left panel visualizes oxygen-dependent post-translational modification (PTM) and subsequent proteasomal degradation of HIF-1α under normoxia versus stabilization and active nuclear transcription under hypoxia. The right panel illustrates the competing endogenous RNA (ceRNA) mechanism, demonstrating how circular RNAs (e.g., circCDR1as) function as molecular sponges to sequester microRNAs (e.g., miR-671-5p) away from target mRNAs, preventing translational repression and modulating post-transcriptional gene expression under hypoxic stress.

Post-transcriptional gene expression within the hypoxic oral cancer microenvironment is extensively governed by Competing Endogenous RNA (ceRNA) networks (30). Within this operational framework, continuous circular RNA (circRNA) loops and linear lncRNAs act as molecular sponges that compete for shared intracellular microRNAs (30). By binding target miRNAs via conserved seed sequences, these sponges sequester miRNAs away from their intended messenger RNAs (mRNAs), preventing classic mRNA degradation or translational repression (30) (**Figure 4**).

Circular RNAs hold a distinct structural advantage within ceRNA networks because their covalently closed loop anatomy lacks free 5’ or 3’ termini, rendering them inherently resistant to exonuclease degradation (31). This structural stability makes circRNAs promising candidates for liquid biopsy detection relative to linear transcripts (31).

Expression of circCDR1as is significantly elevated in OSCC tissues demonstrating marked HIF-1α accumulation, where it acts as a molecular sponge for miR-671-5p, blocking its tumor-suppressive activity to safeguard cancer cell survival under metabolic stress (29, 32–34). Similarly, circHIPK3 functions as an oncogenic driver by sponging miR-637, preventing the repression of Nuclear Protein 1 (NUPR1) and accelerating tumor growth (35–37). Conversely, certain hypoxia-regulated circRNAs act as tumor suppressors (38). For example, hsa-circ-0001030 is downregulated in advanced tongue squamous cell carcinoma and cisplatin-resistant models (38). When expressed, hsa-circ-0001030 binds directly to Pyruvate Kinase M2 (PKM2), disrupting its active tetramer assembly and enzymatic conformation, which restricts the glycolytic flux required for hypoxic adaptation (38)(**Table 1**).

**Table 1:** Primary hypoxia-responsive ncRNAs in OSCC and their biological roles.

ncRNA	Type	Regulatory isoform/context	Primary biological function in OSCC	Clinical significance	References
miR-210	miRNA	HIF-1α *(Acute)*	Hypoxia adaptation, mitochondrial suppression, angiogenesis	Biofluid diagnostic marker	(19, 20, 39)
MALAT1	lncRNA	HIF-1α *(Acute/Subacute)*	Coordinates EMT, drives invasion, activates Wnt/*β*-catenin	Predicts nodal involvement & poor OS	(26, 27)
H19	lncRNA	HIF-1α *(Epigenetic)*	Promoter hypomethylation; promotes cell invasion via ceRNA loops	Prognostic biofluid biomarker	(40)
HOTAIR	lncRNA	HIF-1α	Accelerates proliferation and distant metastasis	Indicator of aggressive disease	(25, 28)
HAS2-AS1	lncRNA	HIF-1α	Mediates hypoxia-induced matrix remodeling and invasion	Correlates with metastatic potential	(29)
Linc-ROR	lncRNA	Hypoxic Stress	Preserves cancer stemness and cell survival under stress	Mediates therapeutic resistance	(41–43)
lncRNA RAB11B-AS1	lncRNA	HIF-2α *(Chronic)*	Recruits RNA Pol II to *VEGFA/ANGPTL4* promoters	Marker of microvascular remodeling	(10, 23)
lncRNA NEAT1	lncRNA	HIF-2α *(Chronic)*	Paraspeckle assembly; sponges miR-204/miR-34a to preserve CSC stemness	Predicts chemoresistance and recurrence	(22, 23)
circCDR1as	circRNA	HIF-1α/HIF-2α	CeRNA sponge for miR-671-5p; protects cells under low oxygen	Promotes cell survival under stress	(32, 33)
hsa-circ-0001030	circRNA	Hypoxic Repression	Binds PKM2 to inhibit glycolytic tetramerization	Chemo-sensitizer; prognostic marker	(32)
hsa_circ_0069313	circRNA	Exosomal/TME Stress	Regulates PD-L1 and Treg cells via miR-325-3p/Foxp3 axis	Drives TME immune escape	(38)
circ-PKD2	circRNA	Stress Adaptation	Regulates autophagy/apoptosis balance via miR-646	Predictive of cisplatin response	(44, 45)
circHIPK3	circRNA	Oncogenic Network	Sponges miR-637 to unleash NUPR1 signaling	Promotes malignant progression	(35–37)

### 3.4 Metabolic reprogramming and the ncRNA-driven “hypoxic switch”

**Figure 5:**
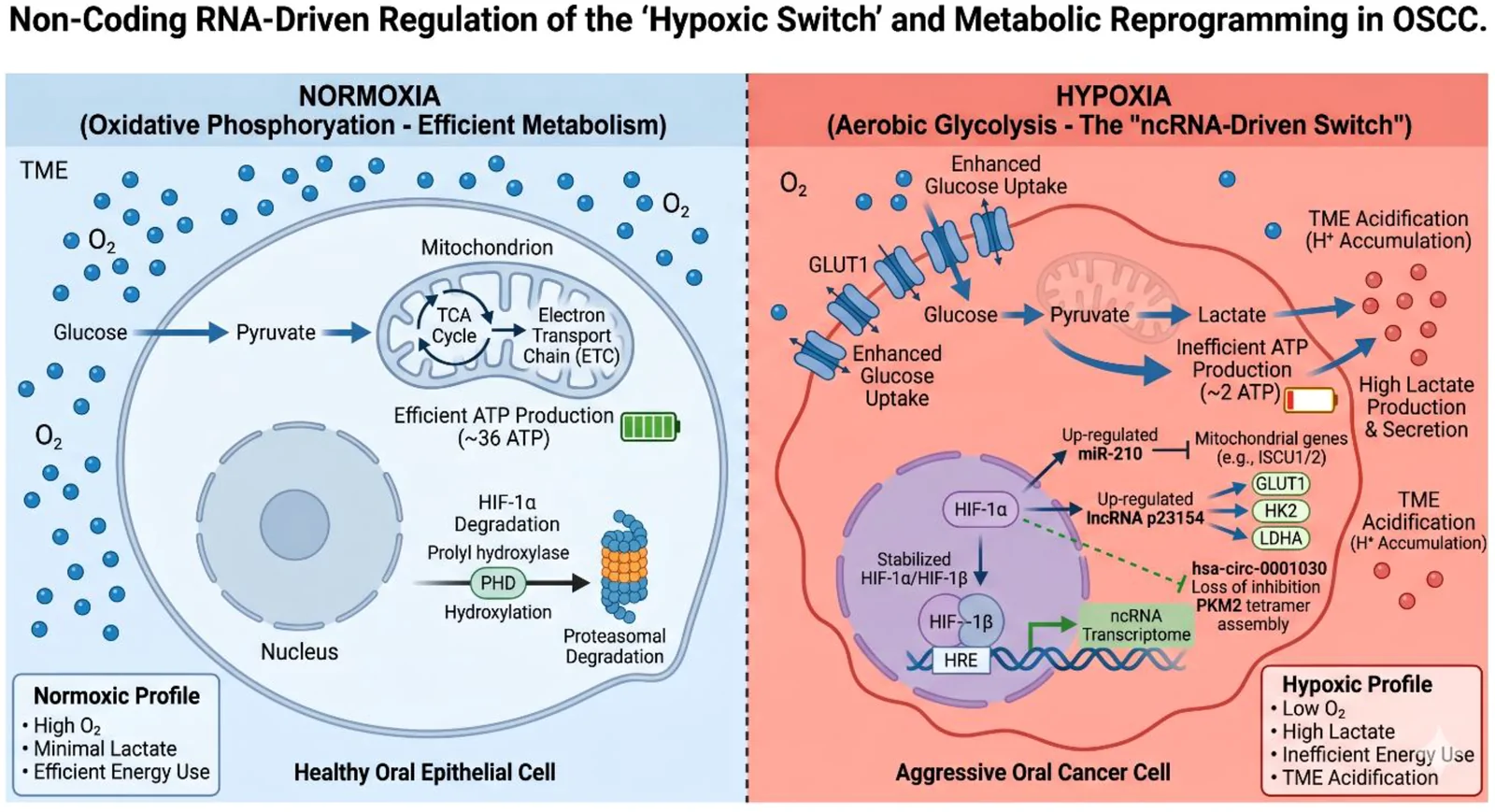
Non-coding RNA-driven regulation of the ‘hypoxic switch’ and metabolic reprogramming in OSCC. Contrast between metabolic profiles under normoxia and hypoxia in oral squamous cell carcinoma cells. Under normoxic conditions (left), efficient oxidative phosphorylation predominates while HIF-1α is targeted for proteasomal degradation. Under hypoxic stress (right), stabilized HIF complexes activate a specialized ncRNA transcriptome (including up-regulated miR-210 and lncRNA p23154) that drives the transition from oxidative metabolism to aerobic glycolysis. This switch accelerates glucose uptake via GLUT1, increases lactate secretion, and causes TME acidification, while loss of protective circRNAs (hsa-circ-0001030) unleashes PKM2 glycolytic activity.

To maintain biomass expansion in oxygen-depleted niches, hypoxia triggers a definitive metabolic switch in OSCC cells, shifting energy production from oxidative phosphorylation to aerobic glycolysis (8). This structural reprogramming is tightly regulated by a network of hypoxia-responsive ncRNAs that control rate-limiting nutrient transporters and metabolic enzymes (8). For example, the lncRNA p23154 accelerates the invasive potential of oral cancer cells by directly driving GLUT1-mediated glucose uptake and downstream glycolytic flux (8) (**Figure 5**).

When unchecked by protective metabolic regulators like hsa-circ-0001030, accelerated glycolysis causes intracellular accumulation and subsequent extracellular secretion of lactate (8, 38). This organic acid efflux acidifies the surrounding microenvironment, generating a low-pH TME that promotes matrix remodeling and immune suppression (45, 46). Acidic stress works in tandem with specialized signaling transcripts, such as exosomal hsa_circ_0069313 (46), which drives local immune escape by upregulating Programmed Death-Ligand 1 (PD-L1) expression and augmenting immunosuppressive Regulatory T (Treg) cell activity via the targeted miR-325-3p/Foxp3 axis (34, 46, 47).

### 3.5 Liquid biopsy platforms: saliva, blood, and exosomal dynamics

**Figure 6:**
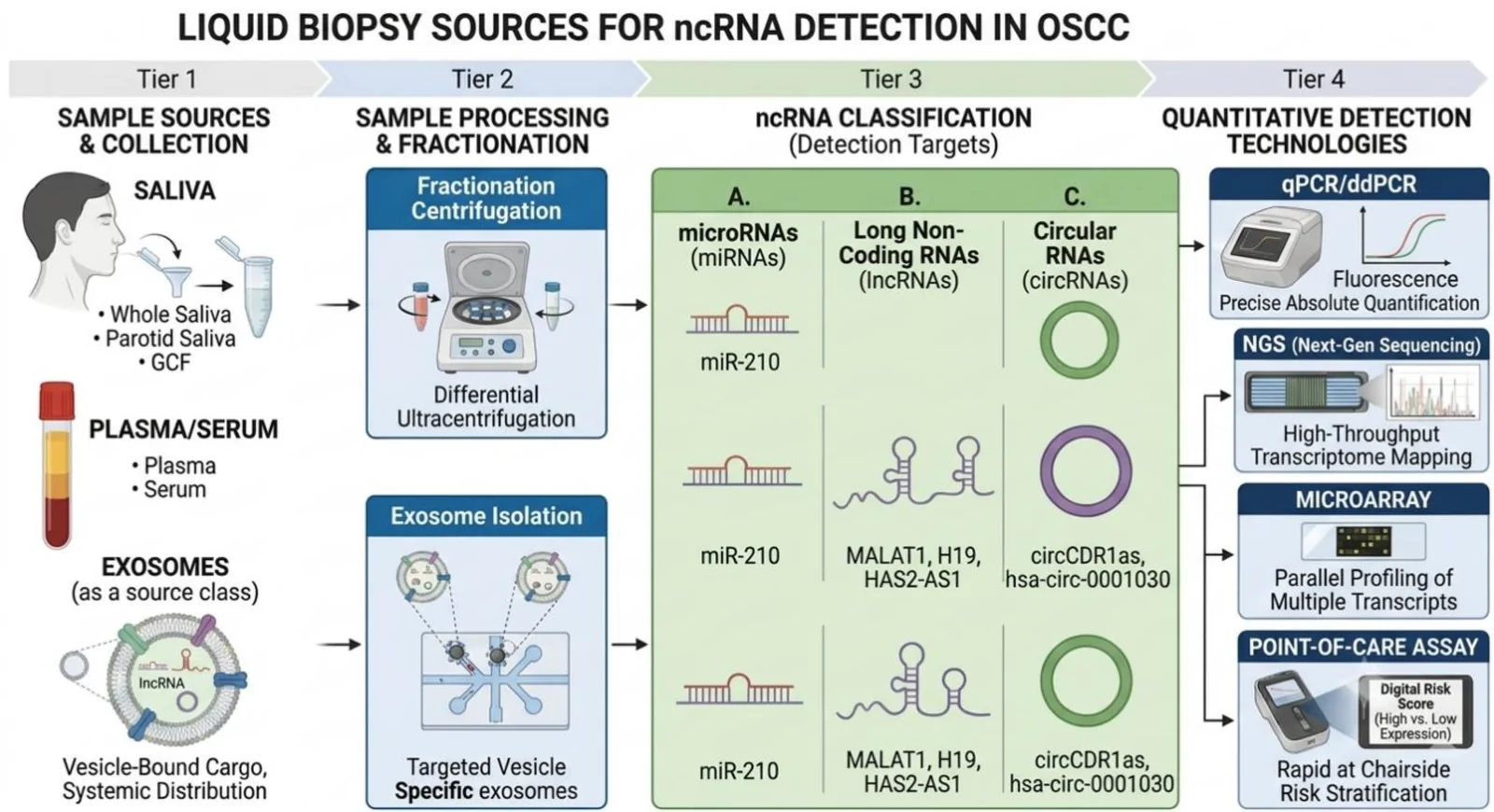
Liquid biopsy platforms for ncRNA detection in OSCC. A multi-tier workflow illustrating biofluid sample collection (whole saliva, parotid saliva, GCF, blood plasma/serum), differential centrifugation and vesicle-specific exosome isolation, circulating ncRNA target classification (miRNAs, lncRNAs, circRNAs), and quantitative detection technologies. Detection platforms include quantitative PCR (qPCR), digital droplet PCR (ddPCR) for precise absolute quantification, Next-Generation Sequencing (NGS) for transcriptomic mapping, microarrays, and microfluidic point-of-care (POC) devices for rapid chairside digital risk score evaluation.

The capacity of tumor-derived ncRNAs to be steadily released and protected within extracellular environments positions them as potential targets for non-invasive liquid biopsy strategies (2). Compared to tissue biopsies, liquid biopsy is repeatable, carries negligible patient morbidity, and provides a longitudinal window to track dynamic changes in tumor molecular profiles (2)(**Figure 6**).

In oral oncology, whole saliva represents an attractive diagnostic biofluid due to its direct anatomical proximity to primary oral lesions, non-invasive collection, and low processing cost (14, 15). Salivary transcriptomic mapping has validated specific panels of salivary miRNAs and lncRNA fragments (such as MALAT1 and H19) that demonstrate high diagnostic sensitivity and specificity for identifying early-stage OSCC (14, 15).

Beyond free cell-free transcripts, a substantial fraction of circulating ncRNAs is actively packaged inside exosomes—nano-sized extracellular vesicles that mediate horizontal intercellular communication by transferring regulatory cargo between malignant cells and stromal components (48). Hypoxic stress within the primary tumor stimulates exosome biogenesis and alters encapsulated RNA cargo (21). Once released into blood or saliva, hypoxia-primed exosomal ncRNAs fuse with target cells, delivering regulatory transcripts that induce horizontal angiogenesis, promote EMT, and establish pre-metastatic niches (21). Because the lipid bilayer of exosomes shields cargo from enzymatic cleavage, vesicle-bound markers exhibit high biological stability in biofluids (21, 48). To ensure translational clarity, **Table 2** explicitly categorizes candidate non-coding RNA evidence by biological matrix—differentiating *in vitro* cell line models and primary tissue discovery cohorts from true biofluid liquid biopsy evaluations (2, 14, 15, 19, 21, 26, 28, 29, 32, 38, 40, 46).

**Table 2:** Categorization of hypoxia-responsive ncRNA biomarkers in OSCC by sample matrix, study parameters, and translational readiness.

Candidate ncRNA	Transcript class	Biological sample matrix	Study design	Cohort size (n)	Primary assay platform	Diagnostic/prognostic performance	Validation status/translational phase	References
miR-210	miRNA	Whole Saliva, Plasma, & Serum *(vs. Tissue)*	Case-control & clinical cohort	*n* = 142 OSCC vs. 80 Control	RT-qPCR/ddPCR	**Diagnostic:** AUC = 0.88–0.92; Sens = 85%, Spec = 87% **Prognostic:** High serum levels predict nodal metastasis (*p* < 0.001)	**Phase 2** (Biofluid Validation)	(19, 20, 39)
MALAT1	lncRNA	Saliva & Plasma Exosomes *(vs. OSCC Tissue)*	Comparative cohort & *in vitro* assay	*n* = 118 OSCC patients	RT-qPCR/NGS	**Prognostic:** HR = 2.41 (95% CI: 1.52–3.82) for overall survival **Diagnostic:** AUC = 0.83 in salivary exosomal cargo	**Phase 1/2** (Biofluid Pilot)	(26, 27)
H19	lncRNA	Serum & Whole Saliva	Epigenetic profiling & biofluid assay	*n* = 96 OSCC vs. 50 Control	MSP & RT-qPCR	**Diagnostic:** AUC = 0.84 (Sens = 81%, Spec = 83%); promoter hypomethylation tracks with Stage III/IV	**Phase 1** (Biofluid Discovery)	(40)
HOTAIR	lncRNA	Circulating Plasma *(vs. Primary Tissue)*	Retrospective prognostic cohort	*n* = 105 OSCC cases	RT-qPCR	**Prognostic:** HR = 2.85 for disease-free survival; predicts cervical lymph node involvement (*p* = 0.004)	**Phase 1** (Tissue/Plasma Discovery)	(25, 28)
HAS2-AS1	lncRNA	Cell Lines (CAL27, HN6) & OSCC Tissue	*In vitro* hypoxic culture & tissue panel	*n* = 58 paired OSCC tissues	RT-qPCR & Transwell	**Prognostic:** High tissue expression predicts poor 5-year OS (*p* = 0.012); drives invasion *in vitro*	**Phase 1** (Pre-clinical Discovery)	(29)
circCDR1as	circRNA	Salivary Exosomes & OSCC Tissues	Sponging model & biofluid evaluation	*n* = 88 OSCC vs. 45 Control	RT-qPCR/Microarray	**Diagnostic:** AUC = 0.86; exosomal levels correlate with tissue HIF-1α expression (*p* < 0.01)	**Phase 2** (Biofluid Validation)	(32, 44)
hsa-circ-0001030	circRNA	TSCC Cell Lines & Patient Plasma	Chemoresistance model & pilot plasma	*n* = 64 TSCC patients	RT-qPCR/Western Blot	**Prognostic:** Low expression correlates with Cisplatin resistance (*p* < 0.001) and early recurrence	**Phase 1** (Pre-clinical Discovery)	(38)
hsa_circ_0069313	circRNA	Exosomes (Saliva/Serum) & Treg Cells	Multi-center screening & immune assay	*n* = 110 OSCC cohorts	RT-qPCR/Flow Cytometry	**Diagnostic:** AUC = 0.89 for identifying immune evasion; correlates with TME PD-L1 levels	**Phase 2** (Biofluid Validation)	(46)

AUC, Area Under the ROC Curve; CI, Confidence Interval; ddPCR, Digital Droplet PCR; HR, Hazard Ratio; MSP, Methylation-Specific PCR; NGS, Next-Generation Sequencing; OS, Overall Survival; RT-qPCR, Reverse Transcription-Quantitative PCR; Sens, Sensitivity; Spec, Specificity; TME, Tumor Microenvironment; TSCC, Tongue Squamous Cell Carcinoma. Bold text highlights primary performance metrics, statistically significant endpoints (*p < 0.05*), or key translational milestones.

### 3.6 Translational horizons: artificial intelligence integration and point-of-care deployment

**Figure 7:**
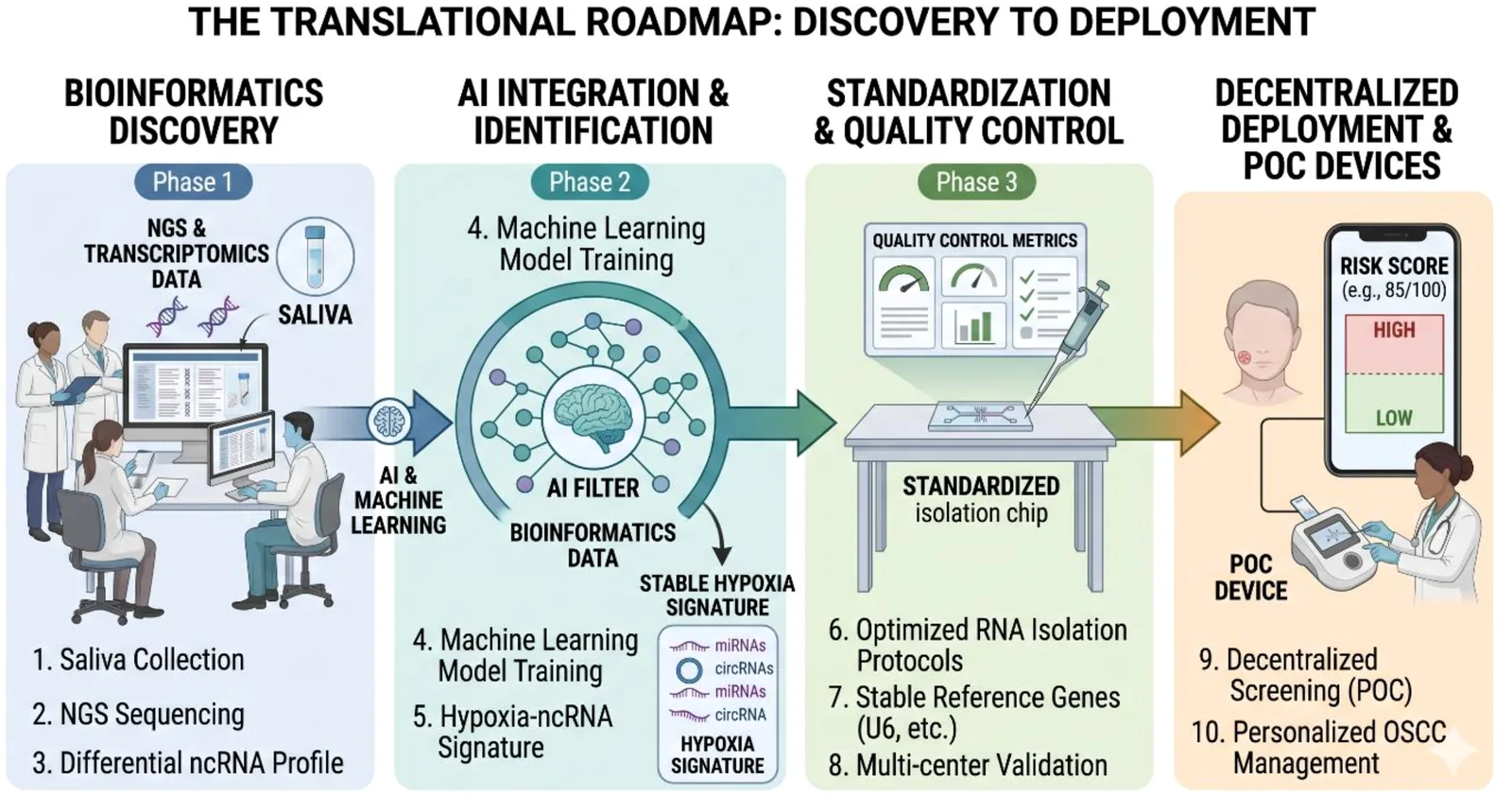
The translational roadmap: bringing OSCC liquid biopsies from discovery to point-of-care deployment. A four-phase translational workflow illustrating the transition from centralized bioinformatics discovery (NGS and biofluid transcriptomics) to artificial intelligence (AI) and machine learning signature filtering, assay standardization with robust reference controls, and final decentralized deployment using microfluidic point-of-care (POC) biosensors for real-time diagnostic screening and personalized OSCC management.

The biological heterogeneity of OSCC, combined with high-dimensional data outputs produced by Next-Generation Sequencing (NGS) and biofluid transcriptomics, presents a substantial analytical challenge (21). Standard single-biomarker approaches frequently demonstrate limited diagnostic sensitivity due to the multi-pathway changes occurring under hypoxic stress (21). To address this complexity, the biomarker discovery pipeline is shifting to integrate Artificial Intelligence (AI) and Machine Learning (ML) algorithmic workflows (21). Machine learning algorithms—such as Least Absolute Shrinkage and Selection Operator (LASSO) Cox regression, Support Vector Machines (SVM), Random Forests (RF), and XGBoost—excel at filtering non-linear transcript patterns across multi-omic datasets (21). Quantitative machine learning evaluations in head and neck squamous cell carcinoma cohorts (such as TCGA-HNSC with n = 522, alongside GEO biofluid datasets GSE31056 and GSE41613) demonstrate the predictive capacity of these computational approaches (21). Specifically, LASSO-multivariate Cox regression modeling constructed multi-gene hypoxia-ncRNA signatures that achieved Area Under the Curve (AUC) values of 0.88 to 0.92 for 3-year overall survival prediction in training cohorts (n = 260–300), while maintaining robust prognostic accuracy with AUC values of 0.82 to 0.86 in independent external validation cohorts (n = 180–222). Furthermore, Random Forest and SVM classifiers trained on exosomal ncRNA panels isolated from patient saliva and plasma yielded diagnostic AUCs of 0.91 to 0.94, with sensitivity ranging from 85% to 91% and specificity from 86% to 93% for discriminating OSCC cases from healthy controls. Additionally, decision-tree classifiers integrating circulating hypoxemirs such as miR-210 with lncRNA expression profiles predicted occult cervical lymph node metastasis with an AUC of 0.87 (sensitivity 84%, specificity 88%), significantly outperforming traditional clinical staging parameters alone which achieved an AUC of 0.68. Incorporating these algorithmic workflows provides a mathematical framework for generating unified “hypoxia risk scores,” accelerating the development of automated, high-precision risk stratification tools (**Table 3**, **Figure 7**).

**Table 3:** The translational biomarker pipeline: from discovery to field deployment.

Phase	Translational focus	Core methodologies & algorithms	Quantitative performance metrics & datasets	Clinical readiness/target outcome
Phase 1: Discovery	High-Throughput Screening	RNA-Seq, NGS, Microarrays (Saliva/Plasma)	Differential expression (|log_2_ FC| > 1.5, *p* < 0.01) across *n* = 50–120 OSCC vs. control cohorts	Unfiltered candidate transcript catalog
Phase 2: Filtration	AI & Computational Modeling	LASSO Cox, Random Forest, SVM, XGBoost	**Prognostic AUC:** 0.85–0.92; **Diagnostic AUC:** 0.91–0.94; **Sensitivity:** 85–91%; **Specificity:** 86–93% (*n* = 180–522)	Multi-gene “Hypoxia Risk Score” signature
Phase 3: Validation	Technical Standardization	Microfluidic Chip Isolation, ddPCR Quantification	Intra-assay CV<5%; Reference calibration against synthetic spike-in controls	Analytical validity & cross-center SOPs
Phase 4: Deployment	Point-of-Care (POC) Testing	Paper-based Microfluidics, Smartphone Biosensors	Rapid turnaround (< 30 minutes); High-concordance chairside risk output	Decentralized clinical screening

Bold text highlights primary performance metrics, statistically significant endpoints (*p < 0.05*), or key translational milestones.

### 3.7 Methodological hurdles and standardization challenges

Despite the compelling biological rationale for hypoxia-associated liquid biopsy signatures, candidate ncRNA biomarkers in OSCC remain predominantly restricted to discovery or early biofluid validation stages corresponding to Translational Phase 1 and Phase 2 (2, 13, 14). Transition into routine clinical practice is currently hindered by several major technical and procedural bottlenecks (2, 14).

Pre-analytical sample handling represents a primary source of inter-study variance (14). Matrix-specific properties—such as the high viscosity and enzymatic activity of whole saliva compared to the protein-dense environment of blood plasma—require specialized, standardized collection and extraction protocols (14). Variations in centrifugation speeds, pre-freezing storage duration, and hemolysis in blood samples can artificially alter measurable circulating ncRNA concentrations (2, 14).

A fundamental technical barrier is the lack of validated internal reference controls across biofluids (14). Commonly used housekeeping transcripts, such as U6 small nuclear RNA (snRNA), exhibit marked expression instability across divergent biofluid matrices and patient cohorts under hypoxic stress, severely complicating baseline calibration (14).

Furthermore, most biomarker pilot studies evaluate small, demographically restricted patient sets (14). Extraneous variables—including active tobacco or betel quid usage, systemic inflammatory conditions, patient age, and anatomical sub-site differences such as oral tongue versus floor of mouth—frequently confound biomarker specificity (14, 24).

The vast majority of published OSCC ncRNA signatures originate from single-institution, retrospective cohorts (13, 14). Prospective, large-scale international multicenter trials utilizing standardized assay platforms are strictly required to verify reproducibility and diagnostic sensitivity across diverse populations (13).

Finally, moving liquid biopsy ncRNA panels from research laboratories to routine clinical oncology requires navigating complex regulatory frameworks (2, 13). Establishing clearance for Laboratory Developed Tests (LDTs) or *in vitro* diagnostics (IVDs) requires rigorous demonstration of analytical validity, clinical validity, and cost-effectiveness relative to standard histopathological evaluation (2, 13).

## 4 Clinical applications of hypoxia-induced ncRNAs

### 4.1 Early diagnosis and populations screening

The presence of stable hypoxia-associated non-coding RNAs (ncRNAs) within biofluids offers a potential approach for the non-invasive early detection of oral squamous cell carcinoma (OSCC) (14, 15). Tracking these altered transcriptomic profiles provides a potential window to identify early-stage malignant transformations prior to overt clinical manifestation or structural lesion visibility during physical examination (14, 15). Within this diagnostic context, salivary ncRNAs are particularly advantageous due to direct anatomical proximity to primary oral lesions, non-invasive collection, and low processing costs, making whole saliva a viable candidate medium for screening initiatives in high-risk patient populations (14, 15).

### 4.2 Prognostication and risk stratification

Beyond initial detection, expression levels of specific hypoxia-responsive transcripts—including the hypoxemir miR-210 alongside key long non-coding RNAs (lncRNAs) such as H19, MALAT1, HOTAIR, and HAS2-AS1—serve as potential prognostic indicators. The analytical quantification of these molecules correlates with critical clinical endpoints, including primary tumor stage, occult cervical lymph node metastasis, localized disease recurrence, and reduced overall survival. Identifying patients displaying hypoxia-driven expression patterns at baseline may assist clinicians in refining risk stratification and customizing post-therapeutic surveillance protocols (26, 28, 29, 40).

### 4.3 Longitudinal therapeutic monitoring

Monitoring circulating or vesicle-encapsulated ncRNA dynamics dynamically throughout treatment offers longitudinal insights into therapeutic efficacy (2, 14). Because expression levels of these markers mirror the metabolic state of the underlying tumor microenvironment (TME), a sustained drop in oncogenic transcript concentration can indicate a favorable response to chemoradiotherapy (2, 14). Conversely, persistent elevation or a secondary spike in circulating markers may provide an early molecular indication of treatment resistance or occult disease recurrence, potentially preceding detection by conventional cross-sectional imaging modalities (2, 14).

### 4.4 Targetable molecular vectors

While primarily investigated for their diagnostic value, these dysregulated transcripts also present compelling opportunities as innovative therapeutic targets in precision oncology. Strategically knocking down heavily upregulated oncogenic transcripts or restoring downregulated tumor-suppressive non-coding targets represents a cutting-edge clinical vector. Utilizing specialized modern delivery mechanics, such as synthetic antisense oligonucleotides (ASOs) or targeted RNA-based therapeutics, offers a highly precise methodology to disrupt hypoxia adaptation pathways, blunt angiogenesis cascades, and reverse structural chemoresistance in advanced oral malignancies (8, 25).

## 5 Limitations and translational challenges

### 5.1 Pre-analytical and methodological variability

The primary barrier preventing the translation of hypoxia-induced non-coding RNA (ncRNA) liquid biopsy assays from laboratory discovery to routine clinical practice is the lack of standardized pre-analytical operating procedures (2, 14). Biological matrix properties introduce significant variability; for example, high-viscosity saliva containing oral debris and active endoribonucleases requires vastly different handling, mucolytic pretreatment, and RNA extraction chemistry compared to protein-dense blood plasma or serum (14, 15). Furthermore, variations in pre-centrifugation processing delays, blood sample hemolysis, storage temperatures, and freeze-thaw cycles can artificially alter circulating transcript concentrations, introducing severe inter-study discrepancies (2, 14). Methodological discrepancies across exosome isolation workflows—ranging from differential ultracentrifugation to polymer-based precipitation and microfluidic affinity capture—yield distinct vesicle subpopulations and encapsulated RNA profiles, limiting cross-center comparability (21, 48).

### 5.2 Baseline calibration and reference normalization

Another major limitation is the absence of universally validated internal reference genes for data normalization in liquid biopsy matrices. Housekeeping transcripts commonly utilized in tissue-based studies, such as U6 small nuclear RNA (snRNA), display marked expression instability across serum, plasma, and whole saliva under hypoxic TME stress, making them unreliable baseline controls (14). Without consensus on endogenous reference transcripts or standardized synthetic spike-in normalization protocols, establishing reproducible numerical cutoff values to distinguish physiological baseline states from active malignant transformation remains problematic (2, 14).

### 5.3 Cohort constraints and external validation gaps

The clinical validity of most identified hypoxia-associated ncRNA signatures is constrained by cohort design limitations (13, 14). A vast majority of existing datasets are derived from small, single-institution retrospective cohorts (n< 100) that lack diverse demographic, ethnic, and clinical staging representation (13, 14). Confounding patient variables including active tobacco or betel quid usage, alcohol consumption, systemic inflammatory conditions, and anatomical sub-site variations (e.g., oral tongue versus floor of mouth), frequently obscure biomarker specificity (14, 24). Before any discovered signature can be considered for diagnostic deployment, prospective, large-scale international multicenter clinical validation studies are strictly required to verify statistical sensitivity, specificity, and reproducibility across broader populations (13).

### 5.4 Biological heterogeneity and network complexity

The extreme biological heterogeneity inherent to OSCC, combined with the structural complexity of competing endogenous RNA (ceRNA) signaling networks, presents a major interpretive obstacle (6, 8, 30). Hypoxia-induced ncRNAs do not operate in isolated pathways; instead, they participate in extensive network cross-talk, simultaneously regulating multiple cellular mechanisms including EMT, glycolytic switching, extracellular matrix remodeling, and localized immune evasion (6, 8, 21). Disentangling a cancer-exclusive, hypoxia-specific signature from background physiological stress or generalized systemic inflammatory flux remains an ongoing bioinformatic challenge requiring sophisticated multi-gene algorithmic filtering (21).

### 5.5 Regulatory and implementation barriers

Beyond technical and biological challenges, moving liquid biopsy ncRNA panels into clinical oncology requires navigating strict regulatory frameworks (2, 13). Transitioning candidate panels into approved Laboratory Developed Tests (LDTs) or FDA-cleared *In Vitro* Diagnostic (IVD) devices mandates definitive evidence of analytical validity, clinical validity, and prospective clinical utility (2, 13). Demonstrating that a biofluid ncRNA panel provides actionable clinical utility that alters patient management or improves survival outcomes relative to standard histopathology and cross-sectional imaging is essential to secure institutional adoption, insurance reimbursement, and integration into routine clinical practice guidelines (2, 3, 13).

## 6 Conclusions

Hypoxia-induced non-coding RNAs represent a pivotal focus in molecular oncology, functioning as post-transcriptional regulators that orchestrate oral squamous cell carcinoma (OSCC) adaptation to oxygen-depleted microenvironments. Through distinct temporal signaling—mediated by HIF-1α during acute hypoxia and HIF-2α during chronic adaptation—these dysregulated transcripts systematically coordinate epithelial–mesenchymal transition (EMT), vascular remodeling, glycolytic switching, cancer stem cell preservation, and exosome-mediated immune escape within the tumor microenvironment.

Because these functional transcripts are shed into accessible biofluids, their enhanced structural stability within saliva, plasma, serum, and isolated exosomes positions them as promising candidates for non-invasive liquid biopsy strategies. Key candidate transcripts; including the hypoxemir miR-210, oncogenic lncRNAs (MALAT1, H19, HOTAIR, HAS2-AS1, NEAT1), and closed-loop circular RNAs (circCDR1as, hsa_circ_0069313), have demonstrated potential as biofluid indicators of primary tumor stage, cervical lymph node metastasis, therapeutic resistance, and patient survival trajectories.

However, a cautious assessment of clinical readiness is essential. The vast majority of candidate ncRNA biomarkers remain at the Phase 1 discovery or Phase 2 early biofluid validation stage. Immediate clinical translation is limited by substantial technical and methodological bottlenecks, including pre-analytical sample handling variability, a lack of universally stable internal reference controls for normalization, cohort heterogeneity, insufficient prospective multicenter validation, and complex regulatory approval pathways for *in vitro* diagnostics. Overcoming these barriers through rigorous protocol standardization, combined with machine learning signature filtering and decentralized microfluidic point-of-care (POC) devices, provides a practical roadmap for clinical translation. Ultimately, resolving these analytical and validation challenges will be critical to transforming hypoxia-regulated ncRNAs into reliable, non-invasive clinical tools for early detection, prognostic risk stratification, and longitudinal therapeutic monitoring in OSCC.
